# Engineering G-quadruplex aptamer to modulate its binding specificity

**DOI:** 10.1093/nsr/nwaa202

**Published:** 2020-08-31

**Authors:** Long Li, Shujuan Xu, Xueyu Peng, Yuzhuo Ji, He Yan, Cheng Cui, Xiaowei Li, Xiaoshu Pan, Lu Yang, Liping Qiu, Jianhui Jiang, Weihong Tan

**Affiliations:** Department of Chemistry and Department of Physiology and Functional Genomics, Center for Research at the Bio/Nano Interface, Health Cancer Center, UF Genetics Institute, McKnight Brain Institute, University of Florida, Gainesville, FL 32611-7200, USA; Department of Chemistry and Department of Physiology and Functional Genomics, Center for Research at the Bio/Nano Interface, Health Cancer Center, UF Genetics Institute, McKnight Brain Institute, University of Florida, Gainesville, FL 32611-7200, USA; Molecular Science and Biomedicine Laboratory (MBL), State Key Laboratory of Chemo/Biosensing and Chemometrics, College of Chemistry and Chemical Engineering, College of Biology, Aptamer Engineering Center of Hunan Province, Hunan University, Changsha 410082, China; Molecular Science and Biomedicine Laboratory (MBL), State Key Laboratory of Chemo/Biosensing and Chemometrics, College of Chemistry and Chemical Engineering, College of Biology, Aptamer Engineering Center of Hunan Province, Hunan University, Changsha 410082, China; Department of Chemistry and Department of Physiology and Functional Genomics, Center for Research at the Bio/Nano Interface, Health Cancer Center, UF Genetics Institute, McKnight Brain Institute, University of Florida, Gainesville, FL 32611-7200, USA; Department of Chemistry and Department of Physiology and Functional Genomics, Center for Research at the Bio/Nano Interface, Health Cancer Center, UF Genetics Institute, McKnight Brain Institute, University of Florida, Gainesville, FL 32611-7200, USA; Molecular Science and Biomedicine Laboratory (MBL), State Key Laboratory of Chemo/Biosensing and Chemometrics, College of Chemistry and Chemical Engineering, College of Biology, Aptamer Engineering Center of Hunan Province, Hunan University, Changsha 410082, China; Department of Chemistry and Department of Physiology and Functional Genomics, Center for Research at the Bio/Nano Interface, Health Cancer Center, UF Genetics Institute, McKnight Brain Institute, University of Florida, Gainesville, FL 32611-7200, USA; Molecular Science and Biomedicine Laboratory (MBL), State Key Laboratory of Chemo/Biosensing and Chemometrics, College of Chemistry and Chemical Engineering, College of Biology, Aptamer Engineering Center of Hunan Province, Hunan University, Changsha 410082, China; Department of Chemistry and Department of Physiology and Functional Genomics, Center for Research at the Bio/Nano Interface, Health Cancer Center, UF Genetics Institute, McKnight Brain Institute, University of Florida, Gainesville, FL 32611-7200, USA; Department of Chemistry and Department of Physiology and Functional Genomics, Center for Research at the Bio/Nano Interface, Health Cancer Center, UF Genetics Institute, McKnight Brain Institute, University of Florida, Gainesville, FL 32611-7200, USA; Department of Chemistry and Department of Physiology and Functional Genomics, Center for Research at the Bio/Nano Interface, Health Cancer Center, UF Genetics Institute, McKnight Brain Institute, University of Florida, Gainesville, FL 32611-7200, USA; Molecular Science and Biomedicine Laboratory (MBL), State Key Laboratory of Chemo/Biosensing and Chemometrics, College of Chemistry and Chemical Engineering, College of Biology, Aptamer Engineering Center of Hunan Province, Hunan University, Changsha 410082, China; Molecular Science and Biomedicine Laboratory (MBL), State Key Laboratory of Chemo/Biosensing and Chemometrics, College of Chemistry and Chemical Engineering, College of Biology, Aptamer Engineering Center of Hunan Province, Hunan University, Changsha 410082, China; Department of Chemistry and Department of Physiology and Functional Genomics, Center for Research at the Bio/Nano Interface, Health Cancer Center, UF Genetics Institute, McKnight Brain Institute, University of Florida, Gainesville, FL 32611-7200, USA; Molecular Science and Biomedicine Laboratory (MBL), State Key Laboratory of Chemo/Biosensing and Chemometrics, College of Chemistry and Chemical Engineering, College of Biology, Aptamer Engineering Center of Hunan Province, Hunan University, Changsha 410082, China; Institute of Molecular Medicine (IMM), Renji Hospital, Shanghai Jiao Tong University School of Medicine, and College of Chemistry and Chemical Engineering, Shanghai Jiao Tong University, Shanghai 200240, China

**Keywords:** G-quadruplex, aptamer, i-motif, binding specificity, cell microenvironment

## Abstract

The use of aptamers in bioanalytical and biomedical applications exploits their ability to recognize cell surface protein receptors. Targeted therapeutics and theranostics come to mind in this regard. However, protein receptors occur on both cancer and normal cells; as such, aptamers are now taxed with identifying high vs. low levels of protein expression. Inspired by the flexible template mechanism and elegant control of natural nucleic acid-based structures, we report an allosteric regulation strategy for constructing a structure-switching aptamer for enhanced target cell recognition by engineering aptamers with DNA intercalated motifs (i-motifs) responsive to the microenvironment, such as pH. Structure-switching sensitivity can be readily tuned by manipulating i-motif sequences. However, structure-switching sensitivity is difficult to estimate, making it equally difficult to effectively screen modified aptamers with the desired sensitivity. To address this problem, we selected a fluorescent probe capable of detecting G-quadruplex in complicated biological media.

## Teaser

The authors have successfully developed allosterically regulated, and binding switchable aptamers from conventional G-quadruplex aptamer that can be readily, economically built, and can achieve more precise recognition of target cells according to the cellular microenvironment.

## INTRODUCTION

Cell surface receptors are membrane-anchored proteins, lipids and carbohydrates that enable cells to communicate with the extracellular matrix. They play key roles in maintaining many vital cellular processes, such as cell growth and cell proliferation [1,2]. Alterations in cell surface receptors represent important molecular signatures of physiological and pathological states. Consequently, over-expressed receptors on disease cells are considered to be disease-related biomarkers, serving as drug targets for effective and safe treatment [3]. However, recent studies suggest that most over-expressed biomarkers are also expressed at a lower or similar level on healthy cells [4,5]. For example, one over-expressed biomarker on the tumor cell surface is nucleolin [6]. It is a phosphoprotein involved in the synthesis and maturation of ribosomes [7], and, as such, it is found on the surface of many normal cells [8]. The existence of the same target receptors on both healthy and diseased cells can lead to unwanted off-target toxicities and serious side effects when applying ligand-drug conjugate [4,9]. This calls for the development of smart structure-switchable ligands able to selectively discriminate between normal and diseased cells.

Antibodies are a class of commonly used ligands for target cell identification, and antibody-drug conjugates (ADCs) have emerged as a popular strategy for targeted drug delivery [10,11]. While promising, antibodies, as recognition moieties, require preparation that is labor-intensive and expensive for protein engineering [12]. Moreover, an abundance of data on building structure-switchable antibodies is hard to find in the literature. On the other hand, aptamers, also termed as ‘chemical antibodies’, are short single-stranded DNA or RNA oligonucleotides that bind with low molecular weight, or macromolecular, analytes with high specificity and affinity [13–18]. To select molecular tools suitable for targeting cancer cells in a complex environment, we have previously reported a cell-based aptamer selection method termed cell-SELEX [19], whereby aptamer selection can be performed without prior knowledge of the molecular signature on the cell surface, thus making it a viable strategy for biomarker discovery [20]. However, aptamers selected in this way still present static binding, whereas dynamic ligands could resolve this problem. More precise recognition of ligands could be achieved by controlling aptameric structure and function in a manner that is responsive to the cellular microenvironment, or, more precisely, tumor microenvironment (TME).

Accordingly, we herein report the development of a single-stranded, structure-switchable aptamer (SW-Apt) able to distinguish diseased from healthy cells by facilitating the binding ability of aptamers to target cells, but suppressing it to non-target cells based on the pH of the cellular microenvironment [21], or TME. The strategy can expand to other cell binding aptamers especially G-quadruplex aptamer by taking advantage of the nucleic acid template nature of aptamers without the need of identifying and replacing structural domains of aptamers [21]. Here, the acidic microenvironment, a well-established TME signal [22], was identified as a proof-of-concept model. In this case, we have utilized DNA i-motifs to achieve the required sensing ability. These tetraplex structures are held together by two parallel-stranded DNAs with multiple cytosine-cytosine+ pairs, and they exhibit high sensitivity to fluctuations in proton concentration [23]. In fact, their excellent pH-sensing property has led to many biomedical applications in fluorescent sensors [24,25], DNA nanomachines [26,27] and logic gates [28]. DNA i-motifs can form multiple G-C interactions with G-quadruplex such that pH-induced structural changes can alter the function of G-quadruplex. Carrying this idea forward, we hypothesized that the DNA i-motif would be a suitable tool for tunable G-quadruplex aptamer design because of (i) high sensitivity towards acidic conditions, in particular the TME; (ii) high folding cooperativity to avoid irreversible duplex of G-rich and C-rich strands; and (iii) potential for tunability of the responsive ranges of SW-Apts. Fundamental scaffolds with deliberate operational characteristics for the rational design of ultrasensitive structure-switchable aptamers are somewhat scarce. However, we demonstrated that the pH-sensitivity of SW-Apts could be modulated by tuning the composition of the i-motif sequence and thus facilitating the binding ability of aptamers to target cells, but suppressing it in non-target cells based on the pH of the TME.

## RESULTS AND DISCUSSION

**Figure 1.**
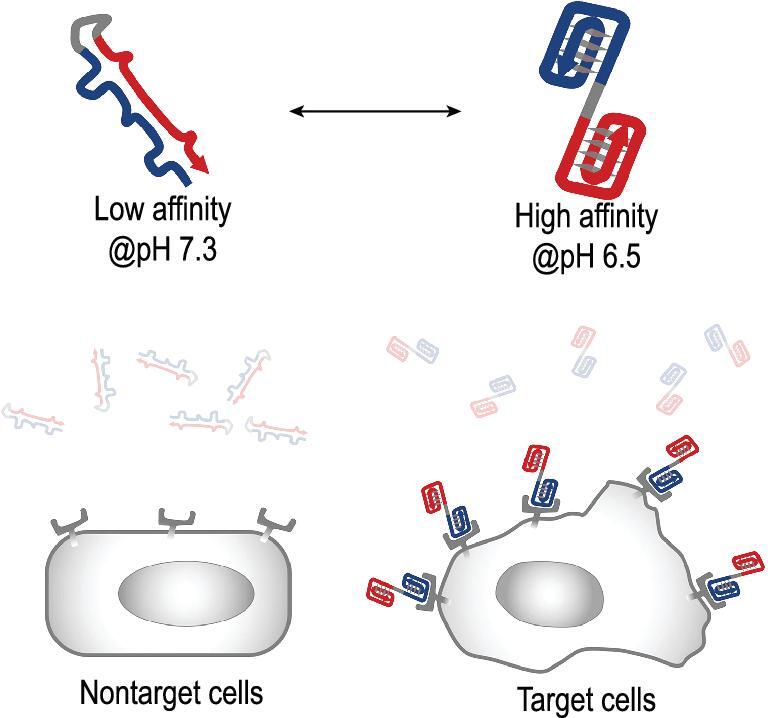
Schematic illustration of double quadruplex structure-switchable aptamer for selective binding of receptors on the surface of target cells.

In this work, we report a series of SW-Apts and the study of their allosteric transition and enhanced recognition of cancer cells. The SW-Apt is composed of double quadruplex structures, including G-quadruplex aptamer as a recognition element, which, as noted above, recognizes nucleolin on the cell surface, and i-motif as a modulating element in the same strand. As shown in Fig. 1, i-motif is added to the 3^′^-end of a G-quadruplex aptamer AS1411 (iAS1411), and i-motif interacts with the G-rich sequence to afford the desired structure-switching operation toward target cell surface receptors in response to both neutral and physiological acidic cellular microenvironments. Because i-motifs can form multiple consecutive CG base pairs with G-quadruplex sequences at physiological pH, the functional conformation of the aptamer is disrupted, thus preventing specific recognition between aptamer and receptor. In contrast, in the acidic microenvironment, both i-motif and G-quadruplex aptamer form simultaneously, allowing the aptamer to regain its targeting ability. We also demonstrated the tunability of SW-Apts from the perspectives of transition range and response sensitivity by just modifying i-motif composition. We wanted to study the transition between duplex and tetraplex forms of the modified aptamer to determine which form would predominate under certain conditions in complex biological media. Therefore, we also identified a light-up fluorescent probe to visualize the formation of the double-quadruplex structure. The simple SW-Apt can be readily and economically constructed, but the degree of its sensitivity cannot be determined, calling for a novel method of ligand screening. The fluorescent probe addresses this problem, showing its potential for screening suitable SW-Apts in a biological environment.

**Figure 2.**
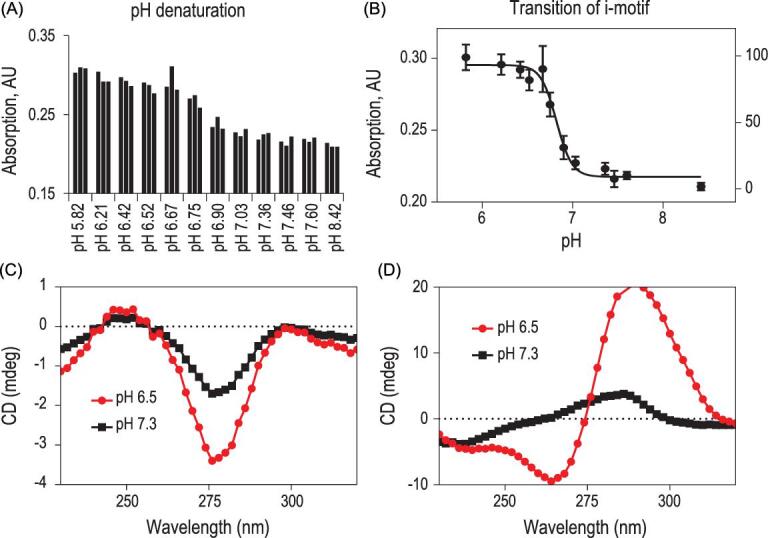
(A, B) UV absorption spectra of iAS1411-1; CD spectra of AS1411 (C) and iAS1411-1 (D) at pH 6.5 (red) and pH 7.3 (black). The measurements were taken at room temperature.

Given the substantial hypochromic effect when i-motif transforms between its folding and unfolding state, we first examined the pH-triggered transitions of an SW-Apt by evaluating the folding and unfolding of i-motif through monitoring the UV absorption at 295 nm. The desired formation of G-quadruplex in i-motif-containing sequence was induced by incubating the samples in buffers with appropriate pH values, followed by comparing the photophysical properties to those of the duplex G-quadruplex-i-motif conjugate. Specifically, aptamer AS1411 and an i-motif sequence with a midpoint of pH 6.8 were used to construct the single-stranded SW-Apt incubated in phosphate-buffered saline (PBS) buffer in the presence of Mg^2+^ to stabilize the formation of a duplex G-rich and C-rich sequence at neutral pH. The buffer used in this step did not affect the photophysical experiment because ions have no absorption at 295 nm. As shown in Fig. 2, UV experiments of the aptamer-i-motif oligonucleotide showed that the intensity of absorption at 295 nm of the ligand remained stable at high or low pH ranges, but decreased upon increasing pH values from 6.67 to 7.03 pH, indicating that the folding cooperativity of i-motif is retained in SW-Apt sequence, which is a prerequisite for using i-motif for aptamer targeting modulation. However, G-quadruplex showed no significant change, indicating that the effect of pH on the absorbance change could be attributed to the transition of i-motif conformation. Circular dichroism (CD) spectroscopy was then used to confirm the pH-triggered duplex and double-quadruplex transformation of the SW-Apt. The pH difference between normal and tumor microenvironments stands at pH 6.5 and pH 7.3, respectively, and these values were used in this study since we aimed at oligonucleotides undergoing structural transition within this range. As shown in Fig. 2B, at pH 6.5, a positive peak was shown in the spectra at 286 nm and a negative peak at 254 nm, which are the characteristic peaks of i-motif, indicating its formation. Importantly, only minimal changes of control CD spectra, G-quadruplex sequence, were observed at the two pH values, confirming that pH changing from 7.3 to 6.5 did not affect the structure of aptamer AS1411, which is essential for aptamer recognition of cell surface receptor. Taken together, these results indicated that i-motif can, indeed, modulate aptamer formation.

**Figure 3.**
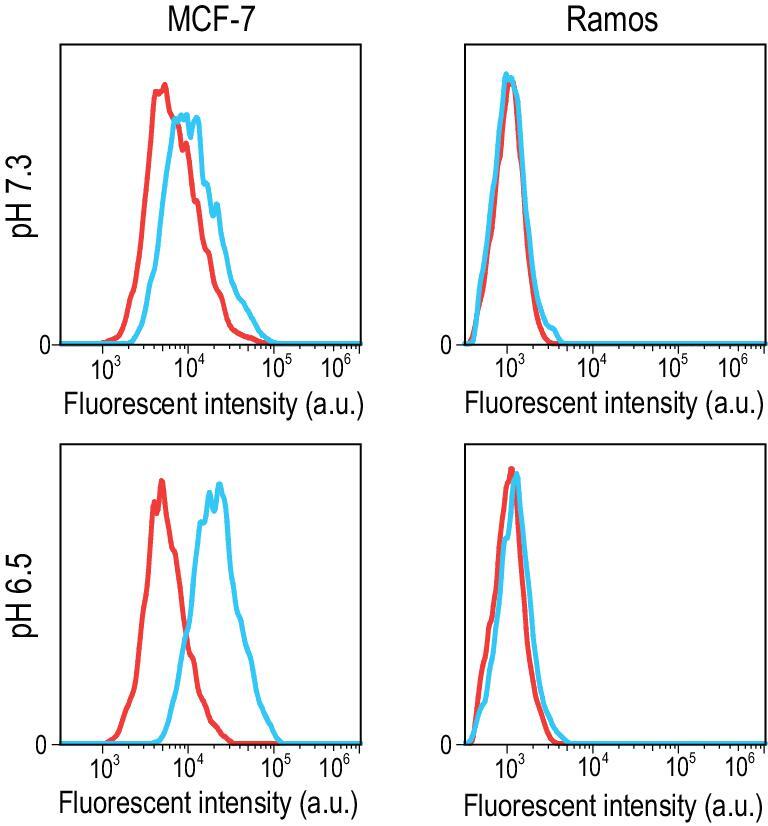
Binding specificity analysis of the iAS1411-1 on MCF-7 cells (left) and Ramos cells (right).

To evaluate whether the modified aptamer retains its targeting capability after engineering i-motif sequence, we studied the capability of targeting MCF-7 cells, which over-express nucleolin on the cell surface, using allosteric regulation of SW-Apt to modulate their binding specificity. To this end, an fluorescein isothiocyanate (FITC) tag was labeled at the 3^′^-end of the modified aptamer (i-motif) to avoid fluorescence quenching by G-base, followed by exposure to MCF-7 cells (a nucleolin-positive cell line) and Ramos cells (a nucleolin-negative cell line), in binding buffer at pH 6.5 and 7.3. Cell surface fluorescence from both cell lines after treatment was measured and compared. As shown in Fig. 3, the modified aptamer could bind MCF-7 cells very well under acidic pH, but not neutral pH, while showing no significant enhancement of fluorescence intensity for negative Ramos cells, suggesting a profound effect from degradation of motif on the G-quadruplex aptamer.

Encouraged by the efficient modulation of aptamer binding ability using i-motif, we next investigated whether the transition range and response sensitivity of the SW-Apt could be tuned to select aptamers with desired properties. The predominant conformation of aptamer-i-motif conjugate at certain pH value is determined by both duplex and quadruplex stability. The fewer G-C base pairs in the duplex, the more likely it is that reduced duplex stability will result, and, hence, less acidic sensitivity. The folding cooperativity of i-motif can be controlled by manipulating its composition. We therefore propose that changing i-motif sequence composition would be instrumental in the control of aptamer response sensitivity, which can be easily synthesized. However, structure-switching sensitivity is difficult to estimate, making it difficult to effectively screen modified aptamers with the desired sensitivity. To address this problem, we selected a fluorescent probe, cyanine dye CyT, capable of detecting G-quadruplex in complicated biological media, as an external probe to visualize the double-quadruplex structure.

**Figure 4.**
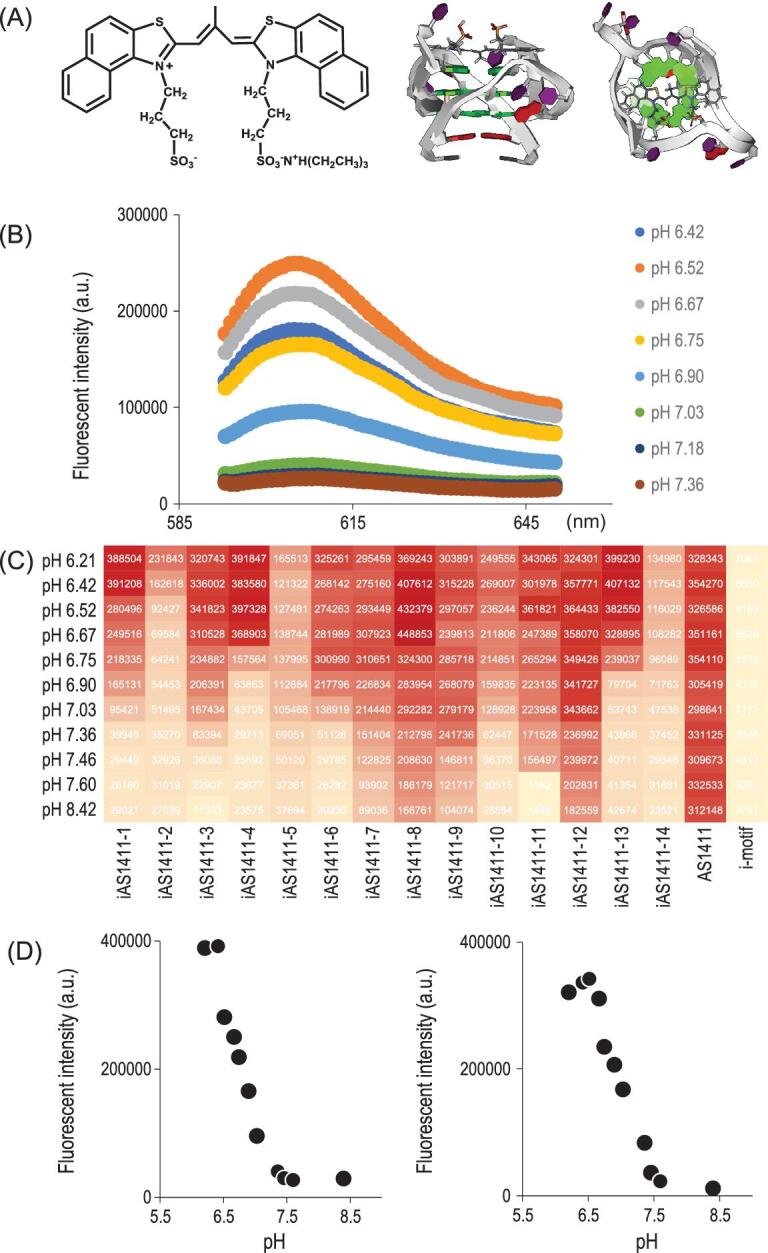
(A) Structure of CyT and binding mode of CyT with DNA G-quadruplex represented as a cartoon. Side view (left side) and axial view (right side); (B) emission of CyT at different pH values; (C) levels of emission for iAS1411-1 to iAS1411-14, AS1411, and i-motif (from left to right) at 610 nm are represented as a heat map; (D) X–y plot chart of emission for iAS1411-1 and iAS1411-3 in (C).

**Figure 5.**
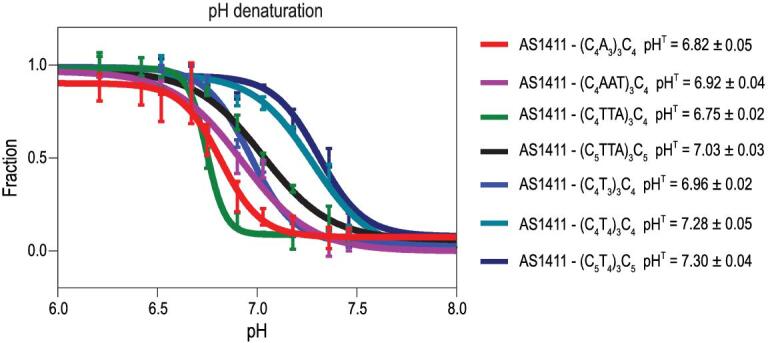
UV analysis showed that the transition midpoint of SW-Apts can be tuned by changing the composition of i-motif (from top to bottom: iAS1411-1, iAS1411-3, iAS1411-4, iAS1411-5, iAS1411-7, iAS1411-8 and iAS1411-9). The experimental points were plotted and fitted with sigmoidal fits. The y axis represented the fraction of folded conformation of SW-Apts.

To confirm the structure switching of G-quadruplex aptamer under different pH values and demonstrate the feasibility of using a small molecular probe for G-quadruplex structure-switching recognition, the fluorescent probe was added into buffer solutions with SW-Apt under different pH values. We compared the fluorescence curves of CyT in the presence of a modified aptamer sequence. In Fig. 4B, the results of emission spectra indicated that CyT could interact with G-quadruplex to induce a very strong emission at 620 nm when diluted in pH 6.2 PBS buffer, while showing a significantly decreased emission at higher pH values, which is consistent with its pH sensitivity, as verified in the UV experiment, suggesting a profound ability of CyT to recognize G-quadruplex in the modified aptamer conjugate upon structure switching. Moreover, we observed minimal fluorescent emission change of CyT against G-quadruplex sequence and i-motif sequence only over a wide pH range, again indicating that CyT can distinguish G-quadruplex from other sequences in a pH-insensitive manner. We then used the fluorescent probe to screen different SW-Apts (iAS1411–1–14) containing the same aptamer sequence, but different i-motif sequences [29], and the changes of CyT emission at 610 nm for the 14 modified aptamers were presented as a heat map, as shown in Fig. 4C. It is obvious that G-quadruplex was present at lower pH, but then gradually disrupted with increasing pH, as indicated by the increasing and decreasing intensities of CyT in the presence of these sequences. The pH sensitivities of modified aptamers were confirmed by photophysical assay (Fig. 5 and Figs S4–S9), yielding results comparable to those of the CyT assay, indicating that the fluorescent probe could effectively visualize the folding and unfolding of the modified G-quadruplex aptamers. This substantially increased the efficiency of screening for SW-Apts with expected pH sensitivity.

**Figure 6.**
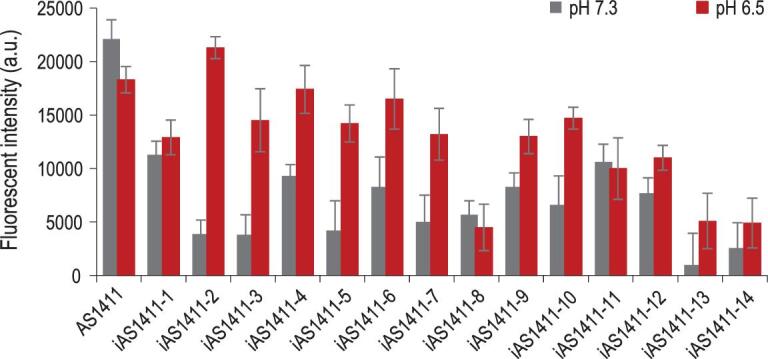
Internalization analysis of aptamer AS1411 and iAS1411-1 to iAS1411-14 (from left to right) to MCF-7 cells, as characterized by flow cytometry.

Having demonstrated the ability of CyT to monitor structure switching of SW-Apts in a buffer based on G-quadruplex-ligand interaction of imaging probe intercalating into G-tetrads, we next studied folding and unfolding of modified aptamers in biological media. The small molecular probe showed extraordinary reliability in detecting G-quadruplex in both a simple buffer solution and a complicated biological environment. All the procedures were the same, except for buffers that were replaced with 10% FBS cell culture media. The CyT emission spectra have been summarized as another heat map shown in Fig. S11. Most acted similarly in both conditions. One shifted to higher pH, possibly because i-motif structure was additionally stabilized in the presence of molecular crowding of cell culture media. There was no obvious change of cell morphology of MCF-7 and Ramos when cultured in media under pH 6.5 and 7.3 for 12, 24 and 48 hours (Fig. S12). Aptamer internalization assays were conducted with those modified aptamers in 10% FBS cell culture media at physiological temperature under pH 6.5 and pH 7.4 for 30 min. According to the data in Fig. 6, most aptamers exhibited higher degrees of internalization at lower pH compared with those at higher pH, indicating the modulation of aptamer function by adding i-motif to aptamer. However, the differences in internalization may be attributed to different degrees of structure disturbances of different i-motifs, or incomplete release of aptamer sequence from duplex, owing to the difference in mid-transition points of those modified aptamers.

## CONCLUSION

In conclusion, we have demonstrated a structure-switching approach to modulate the interaction between aptamer and its target receptor on the cell surface based on conditions of its microenvironment. The SW-Apt can be readily and economically built, and it can be screened by an external probe. Furthermore, we demonstrated that sequence manipulations of i-motif could be utilized through the tuning of SW-Apt folding cooperativity, demonstrating the potential for extension to more G-quadruplex-based functional nucleic acids.

## EXPERIMENTAL PROCEDURES

### DNA synthesis

All DNAs were synthesized using an ABI3400 DNA/RNA synthesizer (Applied Biosystems, Foster City, CA, USA). A, T, G, C and FITC phosphoramidites were purchased from Glen Research (Sterling, VA). The sequences were then purified by reversed-phase HPLC (ProStar, Varian, Walnut Creek, CA) or Urea-PAGE. All samples were quantified using a Bio-300 UV Spectrometer after dissolving in DNA water.

### Cell cultures

MCF-7 cell lines were purchased from American Type Culture Collection (ATCC) and cultured in Dulbecco's modified Eagle medium (DMEM) (Gibco^®^, Life Technologies, Carlsbad, CA) supplemented with 10% fetal bovine serum (FBS, Gibco^®^, Life Technologies, Carlsbad, CA) and 1% penicillin-streptomycin (PS, Life Technologies, Carlsbad, CA). Cells were incubated at 37°C with 5% CO_2_ before use.

### Preparation of binding buffers and serum-containing media

The binding buffer was prepared from Dulbecco's PBS by adding 5 mM MgCl_2_, 2 g/L bovine serum albumin (BSA) (Fisher Scientific) and 100 mg/L yeast tRNA (Sigma-Aldrich, St. Louis, MO, USA). Washing buffer was prepared in the same way, but without BSA. The pH values of PBS, binding buffer, washing buffer and DMEM containing 10% FBS were adjusted to the required values by adding HCl and/or NaOH, using a calibrated pH meter.

### Internalization experiment

The internalization ability of aptamers was determined by incubating cells (1 × 10^6^ cells/mL) with various concentrations of aptamer at 37ºC in culture media (pH 6.5 and 7.3) for 30 min. Cells were washed three times with PBS buffer, suspended in 200 μL of trypsin for 5 min at 37ºC, and then subjected to flow cytometric analysis. The mean fluorescence intensities of the aptamers were obtained for (pH 6.5/pH 7.3) calculation of ratios.

### Fluorescence experiments *in vitro*

Aptamer samples were prepared by diluting to a concentration of 1 μM in PBS at pH 6.5–7.4, heating to 95ºC and cooling slowly (overnight) to room temperature. The measurements were performed on 100 μL aliquots using a 384-well plate after adding 2 μM of CyT and a CLARIOstar microplate reader (BMG LABTECH, Ortenberg, Germany).

### Circular dichroism measurements

CD experiments were performed using a Chirascan™ Circular Dichroism Spectrometer (Applied Photophysics, Surrey, UK). Samples were prepared by slow cooling from 95ºC to room temperature in their corresponding buffers. For each single experiment, 300 μL of a 3 μM sample was used in a 0.3 cm optical path cuvette. Each data point represents the average of three identical scans after a correction for the corresponding buffer blank. The i-motif showed a positive peak at 286 nm at pH 6.5 and a negative peak at 254 nm.

## Supplementary Material

nwaa202_Supplemental_File
