# Supplementary material for: Engineering G-quadruplex aptamer to modulate its binding specificity
Source: Natl Sci Rev. 2020 Aug 31;8(4):nwaa202. doi: 10.1093/nsr/nwaa202 (PMC8065617; doi:10.1093/nsr/nwaa202)
Supplement: nwaa202_Supplemental_File [file nwaa202_supplemental_file.docx]

Supporting Information

**ENGINEERING G-QUADRUPLEX APTAMER TO MODULATE ITS BINDING SPECIFICITY**

Long Li,#† Shujuan Xu,#†‡ Xueyu Peng,‡ Yuzhuo Ji,† He Yan,†‡ Cheng Cui,†‡ Xiaowei Li,† Xiaoshu Pan,† Lu Yang,† Liping Qiu,‡ Jianhui Jiang‡, Weihong Tan†‡$*

†Department of Chemistry and Department of Physiology and Functional Genomics, Center for Research at the Bio/Nano Interface, Health Cancer Center, UF Genetics Institute, McKnight Brain Institute, University of Florida, Gainesville, Florida 32611-7200, USA

‡ Molecular Science and Biomedicine Laboratory (MBL), State Key Laboratory of Chemo/Biosensing and Chemometrics, College of Chemistry and Chemical Engineering, College of Biology, Aptamer Engineering Center of Hunan Province, Hunan University, Changsha 410082, China

$ Institute of Molecular Medicine (IMM), Renji Hospital, Shanghai Jiao Tong University School of Medicine, and College of Chemistry and Chemical Engineering, Shanghai Jiao Tong University, Shanghai 200240, China

Table 4-1. DNA sequences used for double quadruplex structure-switchable aptamer.

| Sequence name | Sequence |
| --- | --- |
| iAS1411-1 | GGTGGTGGTGGTTGTGGTGGTGGTGGTTTCCCCAAACCCCAAACCCCAAACCCC |
| iAS1411-2 | GGTGGTGGTGGTTGTGGTGGTGGTGGTTTCCCCCTTTCCCCCTTTCCCCCTTTCCCCC |
| iAS1411-3 | GGTGGTGGTGGTTGTGGTGGTGGTGGTTTCCCCAATCCCCAATCCCCAATCCCC |
| iAS1411-4 | GGTGGTGGTGGTTGTGGTGGTGGTGGTTTCCCCTTACCCCTTACCCCTTACCCC |
| iAS1411-5 | GGTGGTGGTGGTTGTGGTGGTGGTGGTTTCCCCCAATCCCCCAATCCCCCAATCCCCC |
| iAS1411-6 | GGTGGTGGTGGTTGTGGTGGTGGTGGTTTCCCCCTTACCCCCTTACCCCCTTACCCCC |
| iAS1411-7 | GGTGGTGGTGGTTGTGGTGGTGGTGGTTTCCCCTTTCCCCTTTCCCCTTTCCCC |
| iAS1411-8 | GGTGGTGGTGGTTGTGGTGGTGGTGGTTTCCCCTTTTCCCCTTTTCCCCTTTTCCCC |
| iAS1411-9 | GGTGGTGGTGGTTGTGGTGGTGGTGGTTTCCCCCTTTTCCCCCTTTTCCCCCTTTTCCCCC |
| iAS1411-10 | GGTGGTGGTGGTTGTGGTGGTGGTGGTTTGTTCTTCAATACTACCCCCTTTCCCCCTTTCCCCCTTTCCCCCCTCTCTAGAAGAAC |
| iAS1411-11 | GGTGGTGGTGGTTGTGGTGGTGGTGGTTTGTTCTTCAATCCCCCTTTCCCCCTTTCCCCCTTTCCCCCACTCGAAGAAC |
| iAS1411-12 | GGTGGTGGTGGTTGTGGTGGTGGTGGTTTGTTCTTCCCCCCTTTCCCCCTTTCCCCCTTTCCCCCGAAGAAC |
| iAS1411-13 | GGTGGTGGTGGTTGTGGTGGTGGTGGTTTGTATATAAACCCCTTACCCCTTACCCCTTACCCCCTCTATATAC |
| iAS1411-14 | GGTGGTGGTGGTTGTGGTGGTGGTGGTTTCCCCCTTTTCCCCCACTTATTAAACTCTAATAAGACCCCCTTTTCCCCC |
| i-motif | CCCCAAACCCCAAACCCCAAACCCC |
| AS1411 | GGTGGTGGTGGTTGTGGTGGTGGTGG |


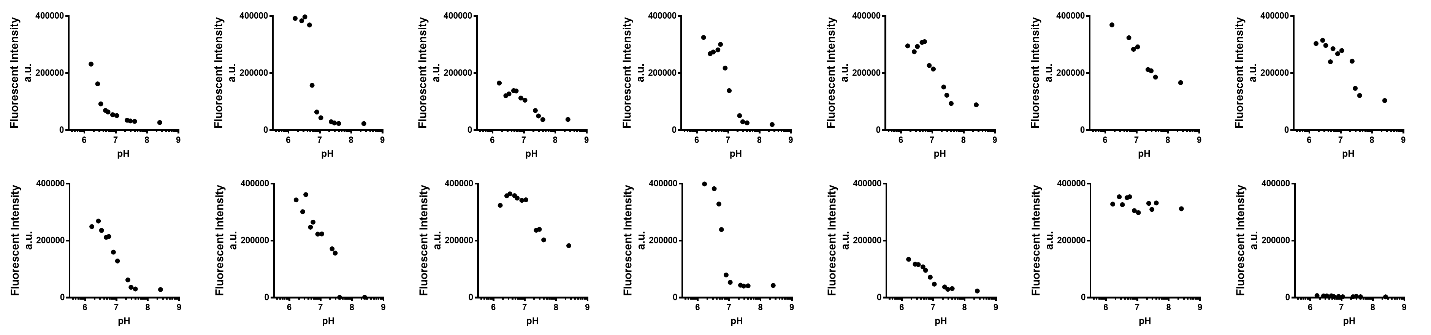


Figure S1. X-y plot chart of emission for iAS1411-2, iAS1411-4 to iAS1411-14, AS1411, and i-motif (from left to right) at 610 nm.


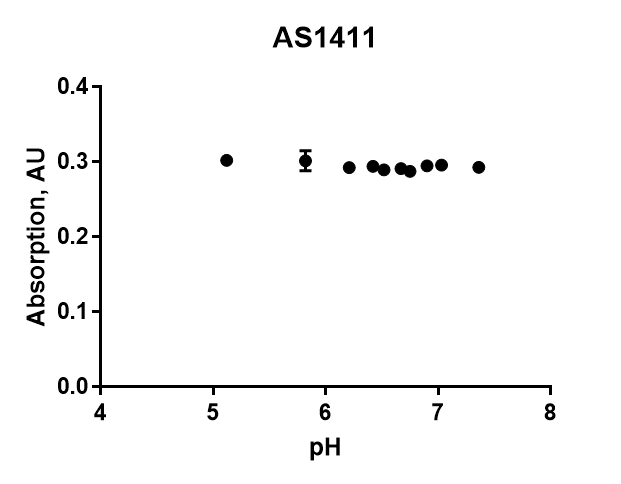


Figure S2. UV absorption spectra of AS1411.

Figure S3. UV analysis showed transition midpoint of iAS1411-2 (midpoint: 6.19 ± 0.06).

Figure S4. UV analysis showed transition midpoint of iAS1411-6 (midpoint: 6.89 ± 0.04).

Figure S5. UV analysis showed transition midpoint of iAS1411-10 (midpoint: 6.95 ± 0.02).

Figure S6. UV analysis showed transition midpoint of iAS1411-11 (midpoint: 6.64 ± 0.0.09).

Figure S7. UV analysis showed transition midpoint of iAS1411-12 (midpoint: 7.06 ± 0.04).

Figure S8. UV analysis showed transition midpoint of iAS1411-13 (midpoint: 6.76 ± 0.06).

Figure S9. UV analysis showed transition midpoint of iAS1411-14 (midpoint: 7.05± 0.03).

Figure S10. CD spectra of AS1411. The i-motif spectra showed a characteristic positive peak at 286 nm and a negative peak at 254 nm at pH 6.5.


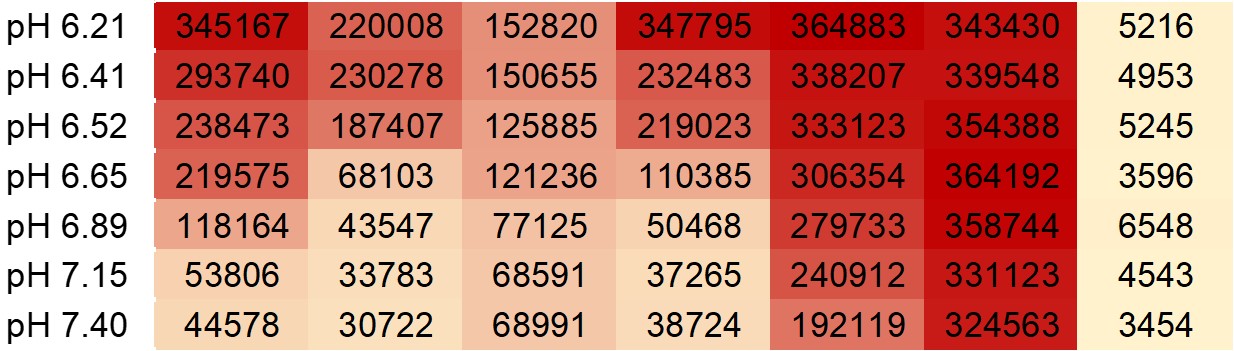


Figure S11. Levels of emission for iAS1411-1, iAS1411-2, iAS1411-3, iAS1411-4, iAS1411-8, AS1411, and i-motif (from left to right) at 610 nm in biological buffer represented as a heat map.


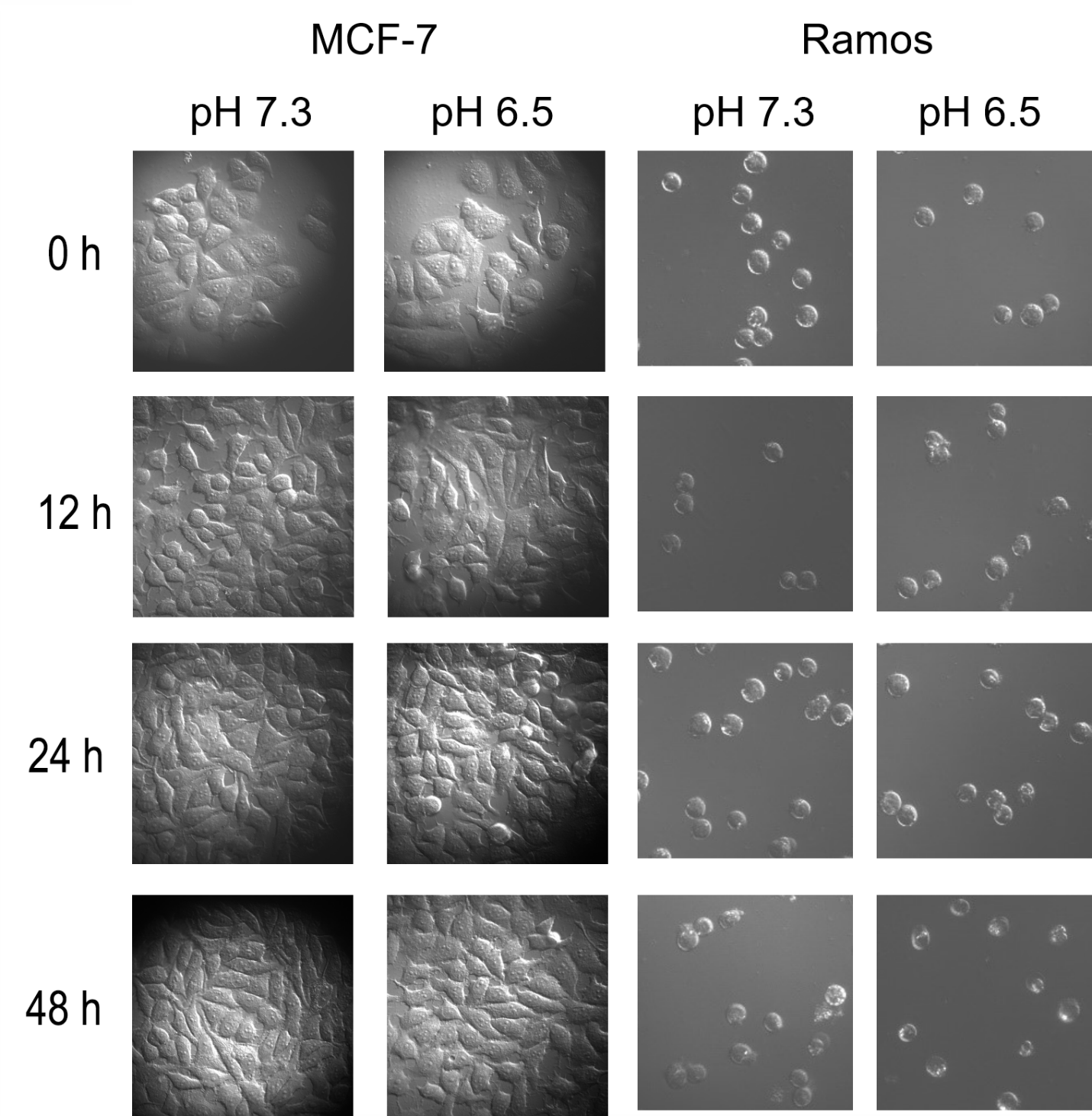


Figure S12. Cell morphology of MCF-7 and Ramos cultured in media under pH 6.5 and 7.3 for 12, 24, and 48 hours.
